# Association of *IFNAR2* rs2236757 and *OAS3* rs10735079 Polymorphisms with Susceptibility to COVID-19 Infection and Severity in Palestine

**DOI:** 10.1155/2023/9551163

**Published:** 2023-09-16

**Authors:** Mohammad Abdelhafez, Abedelmajeed Nasereddin, Omar Abu Shamma, Rajaa Abed, Raghida Sinnokrot, Omar Marof, Tariq Heif, Zaid Erekat, Amer Al-Jawabreh, Suheir Ereqat

**Affiliations:** ^1^Department of Internal Medicine, Faculty of Medicine, Al-Quds University, Abu Deis, East Jerusalem, State of Palestine; ^2^Biochemistry and Molecular Biology Department, Faculty of Medicine, Al-Quds University, Abu Deis, East Jerusalem, State of Palestine; ^3^Al-Quds Bard College, Al-Quds University, East Jerusalem, State of Palestine; ^4^Department of Medical Laboratory Sciences, Faculty of Allied Health Sciences, Arab American University, Jenin, State of Palestine

## Abstract

The clinical course and severity of COVID-19 vary among patients. This study aimed to investigate the potential correlation between the gene polymorphisms of the interferon receptor (*IFNAR2*) rs2236757 and oligoadenylate synthetase 3 (*OAS3*) rs10735079 with the risk of COVID-19 infection and its severity among Palestinian patients. The study was conducted between April and May 2021 on 154 participants who were divided into three groups: the control group (RT-PCR-negative, *n* = 52), the community cases group (RT-PCR-positive, *n* = 70), and the critically ill cases (ICU group; *n* = 32). The genotyping of the investigated polymorphisms was performed using amplicon-based next-generation sequencing. The genotypes distribution for the *IFNAR2* rs2236757 was significantly different among the study groups (*P* = 0.001), while no statistically significant differences were found in the distribution of genotypes for the *OAS3* rs10735079 (*P* = 0.091). Logistic regression analysis adjusted for possible confounding factors revealed a significant association between the risk allele rs2236757A and critical COVID-19 illness (*P*  <  0.025). Among all patients, those who carried the rs2236757GA were more likely to have a sore throat (OR, 2.52 (95% CI 1.02–6.24); *P* = 0.011); the presence of the risk allele rs2236757A was associated with an increased risk to dyspnea (OR, 4.70 (95% CI 1.80-12.27); *P*  <  0.001), while the rs10735079A carriers were less likely to develop muscle aches (OR, 0.34 (95% CI 0.13–0.88); *P* = 0.0248) and sore throat (OR, 0.17 (95% CI 0.05–0.55); *P*  <  0.001). In conclusion, our results revealed that the rs2236757A variant was associated with critical COVID-19 illness and dyspnea, whereas the rs10735079A variant was protective for muscle aches and sore throat.

## 1. Introduction

The coronavirus disease 2019 (COVID-19) is a respiratory and systemic disease caused by severe acute respiratory syndrome coronavirus 2 (SARS-CoV-2), first reported in Wuhan, China, in December 2019, and then rapidly spread across the globe. Worldwide, more than 613 million coronavirus patients were reported with more than 6.5 million total deaths (Worldometers.info: Dover, Delaware, USA) (accessed date 10-09-2022). It is transmitted predominately from person to person mainly through inhalation of small, exhaled respiratory droplets containing infectious virions [1].

The clinical manifestations of COVID-19 vary widely, ranging from asymptomatic to severe and life-threatening acute respiratory distress syndrome (ARDS), multiorgan failure, and ultimately death [2]. The major symptoms of the disease include fever, cough, fatigue, sore throat, headache, and shortness of breath, with progression to pneumonia [3]. Households are favorable venues for viral transmission, where family members may crowd and be in close contact without adhering to social distancing rules or using masks. Although all household contacts of a positive COVID-19 patient are exposed to the virus, not all necessarily get infected. Previous reports showed that male gender, elder age group, and the existence of comorbidities (e.g., cardiovascular, pulmonary, and renal diseases) are risk factors for severe COVID-19 infection [4]. However, the wide range of the reported symptoms suggested that genetic risk factors may also play a crucial role in disease progression. Although the virus's new mutations have emerged (e.g., UK, South Africa, and India), few studies have described interindividual genetic differences in the immune response to these new versions of coronavirus.

It was reported that variants of the angiotensin-converting enzyme 2 (ACE2) gene encode the cellular receptor for SARS-CoV-2, and polymorphisms of serine protease TMPRSS2 affect viral entry and invasion, thereby increasing COVID-19 severity [5, 6]. Moreover, a genome-wide association study (GWAS) conducted in the UK compared the genetic variants in critically ill patients (*n* = 2244) with severe COVID-19 to variants found in a healthy control group. The study revealed significant associations between the severity of COVID-19 and the genetic variants in five loci including chromosome 3p21.31, spanning the *SLC6A20*, *LZTFL1*, *CCR9*, *FYCO1*, *CXCR6*, and *XCR1* genes; chromosome 12q24.13, in the *OAS* gene cluster; chromosome 19p13.2, near tyrosine kinase 2 (TYK2); chromosome 19p13.3, within dipeptidyl peptidase 9 (*DPP9*); chromosome 21q22.1, within the interferon receptor gene *INFAR2*. Of these genes, *IFNAR2* and *OAS* are important in the early stages of the disease, whereas the *DPP9*, *TYK2*, and *CCR2* genes drive inflammatory processes in the late stages of critical COVID-19 [7]. It is well-established that SARS-CoV-2 infection activates innate and adaptive immune responses. The failure of this system and dysregulated massive pro-inflammatory host response would cause harmful tissue damage [8].

The aim of this study was to explore the correlation between four specific SNPs (*IFNAR2* rs2236757, *DPP9* rs2109069, *OAS3* rs10735079, and *LZTFL1* rs73064425 variants) and the susceptibility to COVID-19 infection among Palestinian household contacts. Additionally, the research sought to examine how these SNPs are linked to the clinical manifestations and severity of COVID-19, employing amplicon-based next-generation sequencing (NGS) techniques.

## 2. Materials and Methods

### 2.1. Study Participants

The study participants were recruited from 30 Palestinian families–residing in different cities in the West Bank, Palestine–between April and May 2021. A family was considered eligible for inclusion in the study if it had at least one clinically and laboratory-confirmed COVID-19 case and one laboratory-negative household contact and exhibited no symptoms of COVID-19. We divided all family members into two groups: infected cases (community cases group) and uninfected household contacts (control group). An infected case was defined by having a positive reverse transcription polymerase chain reaction (RT-PCR) test regardless of having symptoms or not and regardless of being a primary or secondary case. An uninfected contact was defined as a family member who had unprotected contact with a positive case, lives at the same place, and stayed asymptomatic for ten days after symptoms onset or RT-PCR diagnosis of a positive case and was tested negative by RT-PCR. Additionally, we consecutively enrolled patients from an intensive care unit (ICU) at the Palestinian Medical Complex, Ramallah-due to critical COVID-19 illness (ICU cases).

We excluded individuals who received COVID-19 vaccination, regardless of the type of the vaccine. Patients' data, including demographic information, symptoms, RT-PCR test results, and comorbidities, were collected via a well-structured questionnaire supervised by healthcare personnel.

### 2.2. Sampling, DNA Extraction, and Genotyping

Blood samples (five ml) were collected in EDTA tubes from all study participants. The DNA was extracted from each blood sample (200 *μ*l) using a genomic QIAamp DNA purification kit as per the manufacturer's instructions (Qiagen, Hilden, Germany) and kept frozen (−20C) for further analysis. All DNA samples were genotyped for the rs2236757 of *IFNAR2*, rs2109069 of *DPP9*, rs10735079 of *OAS3*, and rs73064425 of the *LZTFL1* gene using amplicon-based NGS (NGS). Briefly, two primers (forward and reverse) were used to target each single-nucleotide polymorphism (SNP) as described in Table S1. All primers were modified with over-hanged Illumina adaptor sequences at the 5′ ends (bolded, Table S1), targeting partial sequences of the studied genes. The final product size for each targeted gene is mentioned in Table S1.

The PCR products were visualized by 1.5% agarose gel, cleaned by Agencourt AMPure XP system (X1, A63881; Beckman Coulter Genomics, Indianapolis, IN, USA), and eluted into a final volume of 25 *μ*l. All purified samples were amplified by dual indices PCR to barcode each sample using Nextera XT Index Kit (Illumina, San Diego, CA, USA); five *μ*l from each barcoded sample was pooled together, cleaned again by Agencourt AMPure XP system (X1), and eluted in 50 *μ*l elution buffer. The concentrations of the prepared Libraries were tested by Qubit® Fluorometer (Invitrogen, Carlsbad, CA, USA). A concentration of 4 nM was used with a target of 20k reads for each sample. Deep sequencing was performed by NextSeq 500/550 machine using a 150-cycle Mid Output Kit (Illumina, San Diego, CA, USA).

### 2.3. Bioinformatics and Sequence Analysis

The sequencing data were uploaded to the Galaxy web platform, and the public server at “usegalaxy.org” was used to analyze the obtained DNA sequences [9]. The filtration workflow included Illumina adaptor trim and quality selection of *Q* > 20, with a minimal read length of 100 bp. We used eight virtual probe sequences to identify the targeted variants (Table S1). Ultimately, the genotypes were determined based on the ratio between the read counts for wild-type and minor alleles. SNPs were included in the study if they passed our quality measures: Hardy–Weinberg equilibrium (HWE) >0.05 and genotyping rate >95%.

### 2.4. Statistical Analysis

We performed the statistical analysis using the SPSS package, version 26.0 (SPSS, Inc., Chicago, IL, USA) and the R environment version 4.1.3. All tests were two-tailed, and we considered *P* value <0.05 significant unless specified. We tested for the Hardy–Weinberg equilibrium (HWE) for all SNPs using the “SNPassoc” package [10]. Moreover, we examined the genetic susceptibility of SARS-CoV-2 and the genetic association with the critical COVID-19 illness by comparing the community patients with the controls and the ICU patients with the controls, respectively, using five genetic models (codominant, dominant, over dominant, recessive, and additive). Models were adjusted for: age, gender, smoking, history of hypertension, diabetes mellitus, and coronary artery diseases using the “SNPassoc” package. Adjusted odds ratios (ORs) with the associated 95% confidence intervals (CIs) were calculated for each model. The same models were used to investigate the association of genetic polymorphism with symptoms/signs among ICU and comunity cases groups. The best model for each SNP was selected using the Akaike information criterion [11]. We used Bonferroni correction for multiple comparisons to correct statistical significance (*P*  <  0.05, divided by the number of analyzed SNPs) [12, 13]. Ultimately, we investigated any potential gene-gene interaction.

## 3. Results

### 3.1. Characteristics of Study Participants

A total of 154 Palestinians were included in this study and divided into three groups: COVID-19-infected patients (community cases group, *n* = 70), uninfected household contacts (control group, *n* = 52), and critically ill COVID-19 patients (ICU group, *n* = 32). In each group, the median (IQR) age was 28 (27), 24.5 (21.5), and 61 years (27), respectively. The characteristics and comorbidities of each study group are shown in Table 1. The median age, the prevalence of smoking, diabetes mellitus (DM), hypertension, and coronary artery disease (CAD) were significantly higher in the ICU group (*P*  <  0.05) compared to the community cases and control groups. The clinical characteristics of COVID-19 patients in the community and ICU groups with signs and symptoms frequencies are presented in Table 2. The percentage of symptomatic patients was 93% in the community cases group, whereas 100% in the ICU group. The frequency of fatigability, headache, and loss of taste and/or smell was significantly higher in the community case group (*P*  <  0.05). However, dyspnea and cough were more frequent in the ICU group (*P*  <  0.05).

### 3.2. Genotyping of *IFNAR2* rs2236757 and *OAS3* rs10735079

The minor allele frequency (MAF) of the *IFNAR2* rs2236757A and the *OAS3* rs10735079A was 27% and 50%, respectively. The frequency and genotype distribution of the *IFNAR2* rs2236757A and the *OAS3* rs10735079A among the three study groups are provided in Table 3. The *IFNAR2* rs2236757 genotypes distribution was significantly variable among the study groups (*P* = 0.001), while no significant differences were observed in the distribution of *OAS3* rs10735079 genotypes (*P* = 0.091). The two SNPs, *DPP9* rs2109069 and the *LZTFL1* rs73064425, were excluded from the study due to deviation from the HWE (i.e., *P*  <  0.05) and the low genotyping rate (i.e., <95%).

### 3.3. *IFNAR2* rs2236757 and *OAS3* rs10735079 Polymorphisms and Susceptibility to COVID-19 Infection

Logistic regression analysis under five genetic models adjusted for age, sex, smoking history, DM, hypertension, and CAD was used to investigate the role of *IFNAR2* rs2236757 and *OAS3* rs10735079 polymorphisms in the susceptibility of COVID-19 infection and severity. The community cases group and the ICU group were compared to the control group separately. As shown in Table 4, none of the studied polymorphisms had a statistically significant association with SARS-CoV-2 infection among community cases (*P*  >  0.025) after Bonferroni correction. However, the risk allele rs2236757A of the *IFNAR2* gene was significantly associated with critical COVID-19 illness in all genetic models (*P*  <  0.025) except for the recessive (*P* = 0.4). According to the Akaike information criterion, the dominant model was the best to explain the association (OR, 8.65 (95% CI 1.60–46.68); *P* = 0.005). No significant relationship between the *OAS3* rs10735079 polymorphism and critical COVID-19 illness was observed (*P*  >  0.025; Table S2).

### 3.4. Association of *IFNAR2* rs2236757 and *OAS3* rs10735079 Polymorphisms with COVID-19 Signs and Symptoms

Logistic regression analysis under five genetic models adjusted for age, sex, smoking history, DM, hypertension, and CAD was used to investigate the association of *IFNAR2* rs2236757 and *OAS3* rs10735079 polymorphisms with COVID-19 signs and symptoms.

For all patients (the community cases group and ICU group), the *IFNAR2* rs2236757 GA carriers were more likely to have a sore throat (OR, 2.52 (95% CI 1.02−6.24); *P* = 0.011) (Table 5). In addition, patients who developed dyspnea were more likely to have the risk allele rs2236757A; the association was best explained by the additive model (OR, 4.70 (95% CI 1.80-12.27); *P*  <  0.001). On the other hand, patients with the risk allele rs10735079A were less prone to develop muscle aches (OR, 0.34 (95% CI 0.13–0.88); *P* = 0.0248) and sore throat (OR, 0.17 (95% CI 0.05–0.55); *P*  <  0.001); both associations were best explained by the recessive model (Table 5). Further analyses were performed to investigate the association between genetic polymorphisms and signs and symptoms among community cases and ICU cases seperatly as shown in Table 6. None of the community cases group was homozygous for the risk allele *IFNAR2* rs2236757A (Table 6). However, the risk allele rs2236757A was associated with loss of taste or smell (OR, 3.57 (95% CI 1.19-10.72); *P* = 0.019), muscle aches (OR, 3.65 (95% CI 1.12-11.86); *P* = 0.025), and dyspnea (OR, 4.84 (95% CI 1.45-16.13); *P* = 0.006). We also found that patients with sore throat in the community cases group were unlikely to be homozygous (AA) for the risk allele rs10735079A of *OAS3*; the association was only explained by the recessive model (OR, 0.19 (95% CI 0.05–0.79); *P* = 0.012). Among the ICU group, muscle aches were the only symptoms that had a genetic association; patients with the risk allele rs10735079A were less prone to muscle aches in two models (recessive and additive), and best explained by the additive (OR, 0.22 (95% CI 0.05–0.99); *P* = 0.014) (Table 6). We tested gene-gene interaction in all models that had a significant association with the clinical manifestations; we did not find a statistically significant interaction between the SNPs. However, heterozygous rs2236757 (GA) carriers with sore throat were less likely to be homozygous (AA) for the risk allele rs10735079A (OR, 0.06 (95% CI 0.01–0.57)) (data not shown).

## 4. Discussion

COVID-19 manifestations are variable among patients, even among household members. Before the introduction of COVID-19 vaccination, 33% of people with SARS-CoV-2 infection were reported to be asymptomatic [14]. Wu and McGoogan reported mild COVID-19 infection in 81% of the patients, severe disease in 14%, and critical illness in 5% [15]. Age, comorbidities, sex, and socioeconomic background play an essential role in COVID-19 severity [16–19]. Moreover, novel host genetic factors associated with COVID-19 infection and severity were identified through the collaborated community of human genetics researchers [20].

In the current study, we found that the risk allele *IFNAR2* rs2236757A is significantly associated with critical COVID-19 illness. Such an association was not present for the rs10735079 variant. The rs2236757A variant was found to be related to critical COVID-19 illness in genome-wide significant associations. Additionally, *IFNAR2* has a causal role based on Mendelian randomization result; increased expression of the interferon receptor subunit IFNAR2 reduced the odds of severe COVID-19 (*P* = 0.0043) [7]. Type 1 interferons bind IFNAR2, which leads to activation and signal transduction involving the JAK-STAT pathway [21]. Consequently, this pathway initiates antiviral activity in the target cells and induces apoptosis in infected cells [22]. The role of *IFNAR2* expression was further replicated in other Mendelian randomization studies [23–25]. OAS is a family of antiviral proteins consisting of four members, OAS1, OAS2, OAS3, and OAS-like protein [26]. Both interferon and virus infection stimulate the transcription of *OAS* genes in the cell [27, 28]. Ribonuclease L is activated through the OAS1 to OAS3 proteins; products with 2′-5′ oligoadenylate synthetase activity. Ribonuclease L activation leads to the degradation of the cellular and viral RNA, resulting in the inhibition of protein synthesis and terminating viral replication [29–31]. The variant rs10735079 lies in the interferon-inducible *OAS* gene cluster (*OAS1*, *OAS2*, and *OAS3*) and was associated with critical COVID-19 illness (*P* = 1.65 × 10^−8^) in genome-wide significant associations [7]. However, a similar association was not present in our study.

Different symptom clusters have differences in-hospital outcomes [32]. In a cross-sectional study conducted by Reis et al., involving nearly 60,000 COVID-19 patients, it was found that fever and breathing difficulty significantly increased the likelihood of hospitalization and death. Conversely, runny nose, sore throat, diarrhea, and headache were associated with reduced odds of hospitalization and death. Based on these findings, the researchers concluded that the latter symptoms may indicate a protective effect against severe outcomes from COVID-19 [33]. Sadeghifar et al. used a binary logistic regression model to analyze disease outcomes based on disease symptoms. They showed that shortness of breath and abnormal chest radiographic findings were strong predictors of higher mortality. However, patients with sore throats showed lower mortality rates [34]. Moreover, Chang et al. indicated that body temperature, chills, initial chest X-ray findings, and the presence of diabetes were significant predictors of progression to severe COVID-19 [35].

Studies that evaluate the role of human genetics in the development of different signs and symptoms of COVID-19 are scarce. Williams et al. conducted a study involving 3261 same-sex twins to investigate the presence of heritable components in developing the different symptoms of COVID-19. They found that heritability elements influence certain symptoms, including delirium, diarrhea, fatigue, anosmia, and meal skipping [36].

Herein, we investigated the role of the rs2236757 and the rs10735079 variants in developing the different signs and symptoms of COVID-19 in all COVID-19 patients, and in the community patients and ICU patients, separately. Our results indicated that patients with the risk allele rs2236757A were more likely to have dyspnea and sore throat. Among the community patients, the risk allele rs2236757A was associated with dyspnea, loss of taste or smell, and muscle aches. Surprisingly, patients with the risk allele rs10735079A were unlikely to have a sore throat or muscle aches. In particular, the community cases group was less likely to have a sore throat, and the ICU subgroup was less prone to muscle aches. Given that the risk allele rs10735079A was found to be associated with critical COVID-19 illness in multiple previous studies [7, 20], the inverse association of this risk allele and the presence of a sore throat may indicate that having a sore throat is associated with a lower risk of COVID-19 hospitalization and death, which consistent with the findings of Reis et al. and Sadeghifar et al. [33, 34].

It was reported that interferon-beta inhibits SARS-CoV-2 virus replication in vitro [37]. However, clinical trials did not reveal a clear benefit from interferon therapy for hospitalized patients with severe COVID-19 [38–41]. Yet, a systematic review of five clinical trials concluded that early administration of interferon-beta, combined with other antiviral drugs, is promising [42]. Low expression of *IFNAR2* has a causal role in the progression to critical COVID-19 illness, and our study was in line with the findings of Pairo-Castineira et al., which demonstrated that rs2236757A was associated with the severity of COVID-19 illness [7]. Therefore, a randomized controlled trial that examines the role of interferon therapy in COVID-19 patients who have the rs2236757A variant or other reported variants will help to understand the role of interferon therapy and its benefits.

Our study is limited by the small number of included participants, which was in part due to the newly emerged variants of the SARS-CoV-2 virus. We could not continue recruiting patients when the new SARS-CoV-2 variants became prevalent in Palestine, as different variants may have different manifestations and pathogenesis [43, 44]. Moreover, the COVID-19 vaccination was started by the government, which can influence the severity of COVID-19 disease and SARS-CoV-2 infection [45–47]. The limited number of patients in the present study may explain the lack of association between the rs10735079 polymorphism and the critical COVID-19 illness. Nonetheless, targeting families strengthen the certainty of adequate exposure to the SARS-CoV-2 virus in the control group. Furthermore, the present study is the first in Palestine and one of the limited numbers of studies globally to investigate the role of human genetic factors on signs and symptoms of COVID-19. In conclusion, our study revealed that the *IFNAR2* rs2236757A variant was associated with critical COVID-19 illness. The risk allele rs2236757A was associated with dyspnea and sore throat while patients with the risk allele rs10735079A were less likely to have a sore throat or muscle aches. Our study may provide preliminary results for future genetic association studies aimed at elucidating the role of human genetics in various signs and symptoms of COVID-19 and enhancing our understanding of the pathophysiology of SARS-CoV-2 infection and its complications.

## Figures and Tables

**Table 1:** Baseline characteristics and comorbidities.

	Community patients (*N* = 70)	ICU patients (*N* = 32)	Control (*N* = 52)	*P* value
M : F	32 : 38	19 : 13	33 : 19	0.124
Age^†^	28 (21.5–48.5)	61 (41–68)	24.5 (20.25–41.75)	**<0.001** ^*∗*^
Smoking	15.70%	31.30%	30.80%	0.117
DM	5.70%	25%	3.80%	**0.009**
Hypertension	4.30%	43.80%	3.80%	**0.001**
CAD	4.30%	40.60%	0.00%	**0.001**

^†^Median (25th−75th percentile). ^*∗*^*P* value was obtained by independent-samples Kruskal–Wallis test. *P*  <  0.05 was considered significant. M, male; F, female; DM, diabetes mellitus; CAD, coronary artery disease; ICU, intensive care unit. *P* values below the significance threshold are highlighted in bold.

**Table 2:** Signs and symptoms of COVID-19 in the community cases and the intensive care unit groups.

	Community cases group (*n* = 70)		ICU group (*n* = 32)		*P* value
	*N*	%	*N*	%	
Symptomatic	64	93	32	100	0.118
Runny nose	34	49	7	22	**0.009**
Fatigue	52	75	11	36	**0.001**
Headache	49	71	10	33	**0.001**
Fever	43	62	21	70	0.463
Loss of smell/taste	45	65	12	40	**0.02**
Muscle ache	42	61	21	68	0.51
Diarrhea	24	35	6	19	0.119
Sore throat	27	39	9	30	0.385
Cough	33	49	22	71	**0.037**
Dyspnea	24	35	27	87	**0.001**

*P*  <  0.05 was considered significant. ICU, intensive care unit. *P* values below the significance threshold are highlighted in bold.

**Table 3:** Genotypes distribution of the *IFNAR2* rs2236757 and *OAS3* rs10735079 among the studied groups.

SNP	Alleles (major/minor)	Minor allele frequency *N*(%)	Genotypes	Community cases group	ICU group	Control group	*P* value
rs2236757 (*IFNAR2*)	G/A	83 (26.9)	AA	0	6	2	**0.001**
			GA	35	15	17	
			GG	35	11	33	

rs10735079 (*OAS3*)	G/A	153 (50.3)	AA	20	11	12	0.091
			GA	31	8	28	
			GG	19	13	10	

*P* values were obtained using Pearson's chi-squared test, and a *P*  <  0.05 was considered significant. *P* values below the significance threshold are highlighted in bold.

**Table 4:** Association of *IFNAR2* rs2236757 polymorphism with COVID-19 infection among the studied groups.

SNP	Genetic model	Control	Community cases	ICU	Community cases vs. controls: OR (95% CI); *P*value	ICU group vs. control: OR (95% CI); *P*value
rs2236757 (*IFNAR2*)	Codominant					
	GG	33	35	11	ref	ref
	GA	17	35	15	2.14 (0.92–4.94); 0.028	9.55 (1.53–59.47)
	AA	2	0	6	0 (0.0- NA)	6.41 (0.47–87.80); **0.019**
	Dominant					
	GG	33	35	11	ref	ref
	GA + AA	19	35	21	1.85 (0.82–4.19); 0.134	8.65 (1.60–46.68); **0.005**^†^
	Recessive					
	GG + GA	50	70	26	ref	ref
	AA	2	0	6	0 (0.0- NA); 0.049	2.64 (0.27–26.06); 0.401
	Over dominant					
	GG + AA	35	35	17	ref	ref
	GA	17	35	15	2.29 (1.00–5.26); 0.046	6.99 (1.26–38.86); **0.015**
	Additive					
		52	70	32	1.44 (0.67–3.07); 0.342	3.92 (1.17–13.10); **0.015**

^†^Best model to explain the association according to the Akaike information criterion. The odds ratios and the *P* values were from logistic regression models adjusted for age, gender, smoking history, history of hypertension, diabetes mellitus, and coronary artery disease. After Bonferroni correction, a *P* value <0.025 was considered significant. ICU, intensive care unit; OR, odds ratio; ref, reference; NA, not applicable. *P* values below the significance threshold are highlighted in bold.

**Table 5:** Association of *IFNAR2* rs2236757 and *OAS3* rs10735079 polymorphisms with COVID-19 signs and symptoms in all patients.

rs2236757 (*IFNAR2*)		Yes	No.	OR (95% CI)	*P* value
Sore throat	Codominant^†^				
	GG	13	32	ref	**0.011**
	GA	23	25	2.52 (1.02–6.24)	
	AA	0	6	0.0 (0.0- NA)	
	Dominant				
	GG	13	32	ref	0.124
	GA + AA	23	31	1.98 (0.82–4.76)	
	Recessive				
	GG + GA	36	57	ref	0.026
	AA	0	6	0.0 (0.0- NA)	
	Over dominant				
	GG + AA	13	38	ref	**0.018**
	GA	23	25	2.89 (1.18–7.13)	
	Log additive				
		36	63	1.25 (0.61–2.57)	0.538

Dyspnea	Codominant				
	GG	16	29	ref	**0.002**
	GA	29	20	4.28 (1.57–11.71)	
	AA	6	0	NA	
	Dominant				
	GG	16	29		**0.001**
	GA + AA	35	20	4.81 (1.77–13.09)	
	Recessive				
	GG + GA	45	49	ref	0.054
	AA	6	0	NA	
	Over dominant				
	GG + AA	22	29	ref	**0.009**
	GA	29	20	3.49 (1.32–9.25)	
	Log additive^†^				
		51	49	4.70 (1.80–12.27)	**<0.001**

rs10735079 (*OAS3*)		Yes	No	OR (95% CI)	*P* value

Sore throat	Codominant				
	GG	15	17	ref	**0.004**
	GA	17	20	0.88 (0.33–2.38)	
	AA	4	26	0.16 (0.04–0.58)	
	Dominant				
	GG	15	17	ref	0.099
	GA + AA	21	46	0.47 (0.19–1.16)	
	Recessive^†^				
	GG + GA	32	37	ref	**<0.001**
	AA	4	26	0.17 (0.05–0.55)	
	Over dominant				
	GG + AA	19	43	ref	0.162
	GA	17	20	1.86 (0.78–4.45)	
	Log additive				
		36	63	0.44 (0.24–0.79)	**0.004**

Muscle aches	Codominant				
	GG	22	10	ref	0.079
	GA	27	11	1.11 (0.38–3.22)	
	AA	14	16	0.36 (0.12–1.09)	
	Dominant				
	GG	22	10	ref	0.394
	GA + AA	41	27	0.67 (0.26–1.70)	
	Recessive^†^				
	GG + GA	49	21	ref	**0.0248**
	AA	14	16	0.34 (0.13–0.88)	
	Over dominant				
	GG + AA	36	26	ref	0.191
	GA	27	11	1.81 (0.74–4.47)	
	Log additive				
		63	37	0.60 (0.34–1.05)	0.068

^†^Best model to explain the association according to the Akaike information criterion. The odds ratios and the *P* values were from logistic regression models adjusted for age, gender, smoking history, history of hypertension, diabetes mellitus, and coronary artery disease. After Bonferroni correction, a *P* value <0.025 was considered significant. OR, odds ratio; ref, reference; NA, not applicable. *P* values below the significance threshold are highlighted in bold

**Table 6:** Association of *IFNAR2* rs2236757 and *OAS3* rs10735079 polymorphisms with COVID-19 signs and symptoms in the community patients and ICU patients, separately.

				Yes	No	OR (95% CI)	*P* value
Community patients	rs2236757 *(IFNAR2)*	Loss of taste/smell	Codominant				
			GG	18	17	ref	**0.019**
			GA	27	7	3.57 (1.19–10.72)	
			AA	0	0	NA	
		Muscle aches	Codominant				
			GG	17	18	ref	0.0254
			GA	25	9	3.65 (1.12–11.86)	
			AA	0	0	NA	
		Dyspnea	Codominant				
			GG	8	27	ref	**0.006**
			GA	16	18	4.84 (1.45–16.13)	
			AA	0	0	NA	
	rs10735079 (*OAS3*)	Sore throat	Codominant				
			GG	9	10	ref	0.042
			GA	15	15	1.13 (0.33–3.89)	
			AA	3	17	0.21 (0.04–1.01)	
			Dominant				
			GG	9	10	Ref	0.408
			GA + AA	18	32	0.62 (0.20–1.93)	
			Recessive^†^				
			GG + GA	24	25	ref	**0.012**
			AA	3	17	0.19 (0.05–0.79)	
			Over dominant				
			GG + AA	12	27	ref	0.143
			GA	15	15	2.21 (0.76–6.45)	
			Log additive				
				27	42	0.50 (0.24–1.02)	0.051

ICU patients	rs10735079 (*OAS3*)	Muscle aches	Codominant				
			GG	11	2	ref	0.042
			GA	6	2	0.26 (0.01–5.08)	
			AA	4	6	0.05 (0.00–1.06)	
			Dominant				
			GG	11	2	ref	0.046
			GA + AA	10	8	0.11 (0.01–1.41)	
			Recessive				
			GG + GA	17	4	ref	**0.023**
			AA	4	6	0.11 (0.01–0.91)	
			Over dominant				
			GG + AA	15	8	ref	0.698
			GA	6	2	1.48 (0.20–10.84)	
			Log additive^†^				
				21	10	0.22 (0.05–0.99)	**0.014**

^†^Best model to explain the association according to the Akaike information criterion. The odds ratios and the *P* values were from logistic regression models adjusted for age, gender, smoking history, history of hypertension, diabetes mellitus, and coronary artery disease. After Bonferroni correction, a *P* value <0.025 was considered significant. OR, odds ratio; ref, reference; NA, not applicable. *P* values below the significance threshold are highlighted in bold.

## Data Availability

The data used in the study are available from the corresponding author upon reasonable request.

## Supplementary Materials

Supplementary Materials Table S1: Genes, primers names and sequences, PCR product DNA molecular weight size, and virtual probes sequences that used in sequence analysis. Table S2: Association of *OAS3* rs10735079 polymorphism and COVID-19 infection among families and critical COVID-19 illness.
