# Supplementary material for: Association of IFNAR2 rs2236757 and OAS3 rs10735079 Polymorphisms with Susceptibility to COVID-19 Infection and Severity in Palestine
Source: Interdiscip Perspect Infect Dis. 2023 Sep 16;2023:9551163. doi: 10.1155/2023/9551163 (PMC10517872; doi:10.1155/2023/9551163)
Supplement: Supplementary Materials — Table S1: Genes, primers names and sequences, PCR product DNA molecular weight size, and virtual probes sequences that used in sequence analysis. Table S2: Association of OAS3 rs10735079 polymorphism and COVID-19 infection among families and critical COVID-19 illness. [file 9551163.f1.docx]

**Supplemental Materials**

Table S1: Genes, Primers names and sequences, PCR product DNA molecular weight size, and virtual probes sequences that used in sequence analysis

Table S2: Association of *OAS3* rs10735079 polymorphism and COVID-19 infection among families and critical COVID-19 illness

**Table S1:** Genes, Primers names and sequences, PCR product DNA molecular weight size, and virtual probes sequences that used in sequence analysis

| Gene | Oligo name | Sequence | PCR product size/bp | Target SNP:Virtual probe |
| --- | --- | --- | --- | --- |
| *DPP9* | rs2109069F | **TCGTCGGCAGCGTCAGATGTGTATAAGAGACAG**CTCCAGCCTGGGTAACAGAG | 220 | A:GGGGAGTGGAA |
|  | rs2109069R | **GTCTCGTGGGCTCGGAGATGTGTATAAGAGACAG**TTTTGGGCTCCAAGAGACTG |  | G: GGGGAGTGGAG |
| *OAS3* | rs10735079F | **TCGTCGGCAGCGTCAGATGTGTATAAGAGACAG**CATCTGGGAGTTTCCCTCAG | 250 | A: GGGCCTGGGGACA |
|  | rs10735079R | **GTCTCGTGGGCTCGGAGATGTGTATAAGAGACAG**CGAAGGAGAACACAAAAATCA |  | C: GGGCCTGGGGACC  G: GGGCCTGGGGACG |
| *IFNAR2* | rs2236757F | **TCGTCGGCAGCGTCAGATGTGTATAAGAGACAG**CCTTTTTCTTACCAAGCCTGTG | 290 | A: ACCTCTAAATG |
|  | rs2236757R | **GTCTCGTGGGCTCGGAGATGTGTATAAGAGACAG**GCTTGCTCATCACTGTGCTC |  | G: GCCTCTAAATG |
| *LZTFL1* | rs73064425F | **TCGTCGGCAGCGTCAGATGTGTATAAGAGACAG**AGCCACTGAATGTGGCCTAT | 270 | C: AAATGACAAAAATTAAC |
|  | rs73064425R | **GTCTCGTGGGCTCGGAGATGTGTATAAGAGACAG**GTTGCAGTGAGCCAAGATCA |  | T: AAATGACAAAAATTAAT |

**Table S2:** Association of *OAS3* rs10735079 polymorphism and COVID-19 infection among families and critical COVID-19 illness

| **SNP** | **Genetic model** | **Control** | **Community cases** | **ICU cases** | **Community cases Vs. Control: Adjusted OR (95% CI); Adjusted P-value** | **ICU cases Vs. Control: Adjusted OR (95% CI); Adjusted P-value** |
| --- | --- | --- | --- | --- | --- | --- |
| ***rs10735079*** (*OAS3*) | **Codominant** |  |  |  |  |  |
|  | GG | 10 | 19 | 13 | Ref  0.50 (0.19-1.36)  0.89 (0.29-2.76); 0.285 | Ref  0.22 (0.04-1.17)  0.52 (0.10-2.85); 0.185 |
|  | GA | 28 | 31 | 8 |  |  |
|  | AA | 12 | 20 | 11 |  |  |
|  | **Dominant** |  |  |  |  |  |
|  | GG | 10 | 19 | 13 | Ref  0.61 (0.24-1.57); 0.3 | Ref  0.32 (0.08-1.37); 0.122 |
|  | GA+AA | 40 | 51 | 19 |  |  |
|  | **Recessive** |  |  |  |  |  |
|  | GG+GA | 38 | 50 | 21 | Ref  1.43 (0.58-3.50); 0.433 | Ref  1.21 (0.28-5.19); 0.795 |
|  | AA | 12 | 20 | 11 |  |  |
|  | **Overdominant** |  |  |  |  |  |
|  | GG+AA | 22 | 39 | 24 | Ref  0.53 (0.24-1.17); 0.116 | Ref  0.31 (0.08-1.28); 0.094 |
|  | GA | 28 | 31 | 8 |  |  |
|  | **Additive** |  |  |  |  |  |
|  |  | 50 | 70 | 32 | 0.96 (0.56-1.67); 0.896 | 0.70 (0.29-1.72); 0.432 |

The odds ratios and the P values were from logistic regression models adjusted for age, gender, smoking history, history of hypertension, diabetes mellitus, and coronary artery disease. After Bonferroni correction a P-value < 0.025 was considered significant. ICU, intensive care unit; OR, odds ratio; Ref, reference.
